# The H9N2 avian influenza virus increases APEC adhesion to oviduct epithelia by viral NS1 protein-mediated activation of the TGF-β pathway

**DOI:** 10.1128/jvi.01512-23

**Published:** 2024-02-28

**Authors:** Jinjie Han, Wenchi Chang, Junyang Fang, Xiaolan Hou, Zhijun Li, Jingyu Wang, Wen Deng

**Affiliations:** 1 College of Veterinary Medicine, Northwest A&F University, Yangling, Shaanxi, China; Emory University School of Medicine, Atlanta, USA

**Keywords:** H9N2, influenza A, APEC, salpingitis, TGF-β

## Abstract

H9N2 avian influenza is a low-pathogenic avian influenza circulating in poultry and wild birds worldwide and frequently contributes to chicken salpingitis that is caused by avian pathogenic *Escherichia coli* (APEC), leading to huge economic losses and risks for food safety. Currently, how the H9N2 virus contributes to APEC infection and facilitates salpingitis remains elusive. In this study, *in vitro* chicken oviduct epithelial cell (COEC) model and *in vivo* studies were performed to investigate the role of H9N2 viruses on secondary APEC infection, and we identified that H9N2 virus enhances APEC infection both *in vitro* and *in vivo*. To understand the mechanisms behind this phenomenon, adhesive molecules on the cell surface facilitating APEC adhesion were checked, and we found that H9N2 virus could upregulate the expression of fibronectin, which promotes APEC adhesion onto COECs. We further investigated how fibronectin expression is regulated by H9N2 virus infection and revealed that transforming growth factor beta (TGF-β) signaling pathway is activated by the NS1 protein of the virus, thus regulating the expression of adhesive molecules. These new findings revealed the role of H9N2 virus in salpingitis co-infected with APEC and discovered the molecular mechanisms by which the H9N2 virus facilitates APEC infection, offering new insights to the etiology of salpingitis with viral-bacterial co-infections.

**IMPORTANCE:**

H9N2 avian influenza virus (AIV) widely infects poultry and is sporadically reported in human infections. The infection in birds frequently causes secondary bacterial infections, resulting in severe symptoms like pneumonia and salpingitis. Currently, the mechanism that influenza A virus contributes to secondary bacterial infection remains elusive. Here we discovered that H9N2 virus infection promotes APEC infection and further explored the underlying molecular mechanisms. We found that fibronectin protein on the cell surface is vital for APEC adhesion and also showed that H9N2 viral protein NS1 increased the expression of fibronectin by activating the TGF-β signaling pathway. Our findings offer new information on how AIV infection promotes APEC secondary infection, providing potential targets for mitigating severe APEC infections induced by H9N2 avian influenza, and also give new insights on the mechanisms on how viruses promote secondary bacterial infections in animal and human diseases.

## INTRODUCTION

Influenza A viruses are the causing agent of human seasonal influenza, and IAVs could also circulate among animal hosts including birds and mammals, with the risk for emerging of novel virus strains causing human endemic or pandemic (1, 2). The avian influenza virus (AIV) subtype of avian influenza A (H9N2), classified as low-pathogenic avian influenza based on molecular features and pathological typing (3), has been circulating widely in poultry and human in Asian countries since the mid-1990s, with mortality rates ranging from 5% to 30% in chickens (4). Our previous study identified that the oviducts are one of the potential targets for the H9N2 virus (5), which replicates at high titers in the oviduct of avian species, resulting in salpingitis and reduced egg production and eggshell thickness (6).

H9N2 infection has also been found to exacerbate *Escherichia coli* (*E. coli*) proliferation in the digestive tract in chicken and lead to more severe lung damage in pneumonia in a mouse model (7, 8), but whether H9N2 AIV contributes to avian salpingitis caused by *E. coli* is still unknown. The avian pathogenic *Escherichia coli* (APEC) is an extraintestinal *Escherichia coli* pathogen that causes a variety of local and systemic infections in birds, ducks, and other avian species including *Gallus* in this study (9). Colibacillosis is one of the leading causes of mortality (up to 20%) and can also lead to the occurrence of many inflammations, such as airsacculitis and egg peritonitis. APEC infection appeared to have a greater impact on peak egg production and late lay period chickens than on uninfected healthy laying peak period hens, causing severe salpingitis, a decrease in egg production, and having zoonotic potential (10–12). In fact, APEC is the most common bacterial pathogen attacking chickens, causing hundreds of millions of dollars in economic losses to the global poultry industry (13).

APEC leads to systemic infections in chickens or as a secondary infection to viral infections, such as infectious bronchitis, Newcastle disease, and AIV. It can infect the host through the mouth and respiratory tract and can also be transmitted vertically through infected eggs (14, 15). APEC utilizes various virulence and pathogenic factors to cause chicken diseases, mainly adhesin, invasion, and protection. These factors promote APEC adhesion, invasion, and systemic dissemination, thereby establishing infection in chickens (16). However, the mechanism of secondary infection of APEC after H9N2 and the co-infection of two causes of salpingitis is still unclear.

The purpose of this study was to investigate the mechanism of increased APEC adhesion after H9N2 infection of fallopian tube epithelial cells and the effect of co-infection on inflammatory factors and inflammatory pathways. To achieve this goal, H9N2 AIV-enhanced APEC infection was investigated *in vitro* and *in vivo*, and H9N2 virus-induced changes of expression of adhesive molecules on cell surface was studied to reveal the protein responsible for APEC adhesion. Furthermore, cell signaling pathways involved in the regulation of the adhesive molecules were investigated, and the underlying molecule mechanisms by which the viral protein activates adhesive molecules was discovered. This study reveals the roles and mechanisms of H9N2 viruses in H9N2 AIV and APEC co-infection, offering new insights to the etiology of salpingitis with virus and bacteria co-infections.

## RESULTS

### H9N2 infection caused adhesion molecule alterations and enhanced APEC adherence to chicken oviduct epithelial cells

**Fig 1:**
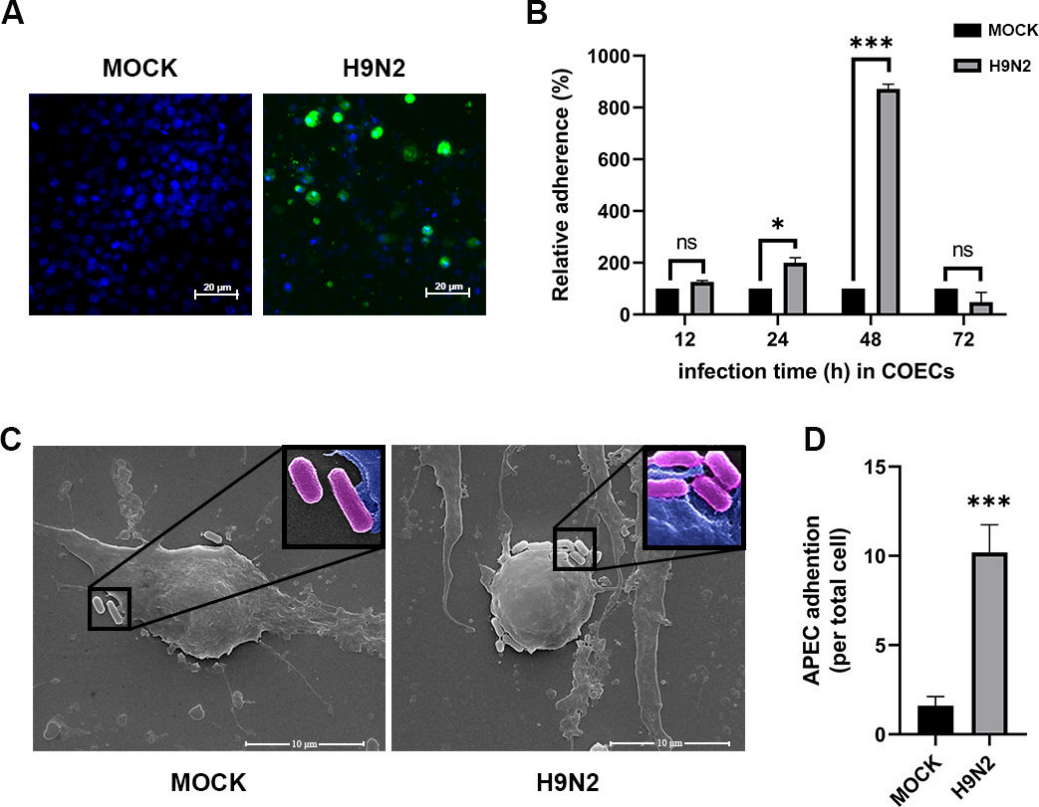
H9N2 infection enhances APEC adhesion to COECs. (**A**) Infectivity of H9N2 virus to COECs confirmed by immunofluorescence staining of NS1 48-h post-infection. (**B**) Relative adherence of APEC to COECs in the presence of H9N2 AIV infection. COECs were infected with H9N2 AIV (H9N2) with an MOI of 1 or mock-infected (MOCK) for 12, 24, 48, and 72 h, and adhesion bacterial cells were counted by plate count method. All data were normalized by the mock-infected groups, which were considered as 100%. (**C**) Representative SEM images of APEC adhered to COECs. COECs were infected with H9N2 for 48 h, followed by an adhesion assay with APEC at a CFU of 1 × 10^8^. Scale bars are equal to 10 µm. (**D**) Statistical analysis of the APEC adhering to COECs. Numbers of bacteria attaching to COECs were counted, and three fields of view were analyzed for each group. The data represent the mean ± standard deviation of three independent experiments. *P* values were calculated using two-tailed Student’s *t*-test. An asterisk indicates a significant difference in comparison to the indicated control. *P < 0.05, ***P < 0.001. ns, not significant.

To understand the potential mechanisms on the increased APEC infection after H9N2 virus infection, we first used an *in vitro* model to investigate the potential impact of H9N2 infection on APEC adhesion to chicken oviduct epithelial cells (COECs). Infectivity of the H9N2 virus to COECs was confirmed by immunofluorescence assay detecting the NS1 protein of the virus. When inoculated at a multiplicity of infection (MOI) of 1, about 80% of the cells showed positive staining for the viral protein NS1 at 48 h post-infection, indicating successful infection of COECs by the H9N2 virus (Fig. 1A). Then, APEC were inoculated in COECs with co-infected H9N2 or mock infection, and APEC adhered to the cells were measured by plate counting at different time points after co-infection. As shown in Fig. 1B, at 12 h after inoculation, there was no significant difference on the APEC adhesion between the H9N2 infected and the mock infected group, but significant higher numbers of adhered APEC were observed in H9N2 infected cells at 24 and 48 h after APEC inoculation, with about eight times higher APEC adhesion in H9N2 infected cells than that of control cells at 48 h after co-infection. APEC adhesion was further checked by scanning electron microscopy 48 h after co-infection, and significant higher numbers (about 10 times) of APEC were also observed in H9N2-infected COECs than in the control, consistent with the plate counting data (Fig. 1C and D). These results demonstrated H9N2 infection facilitates APEC adhesion to cultured COECs.

**Fig 2:**
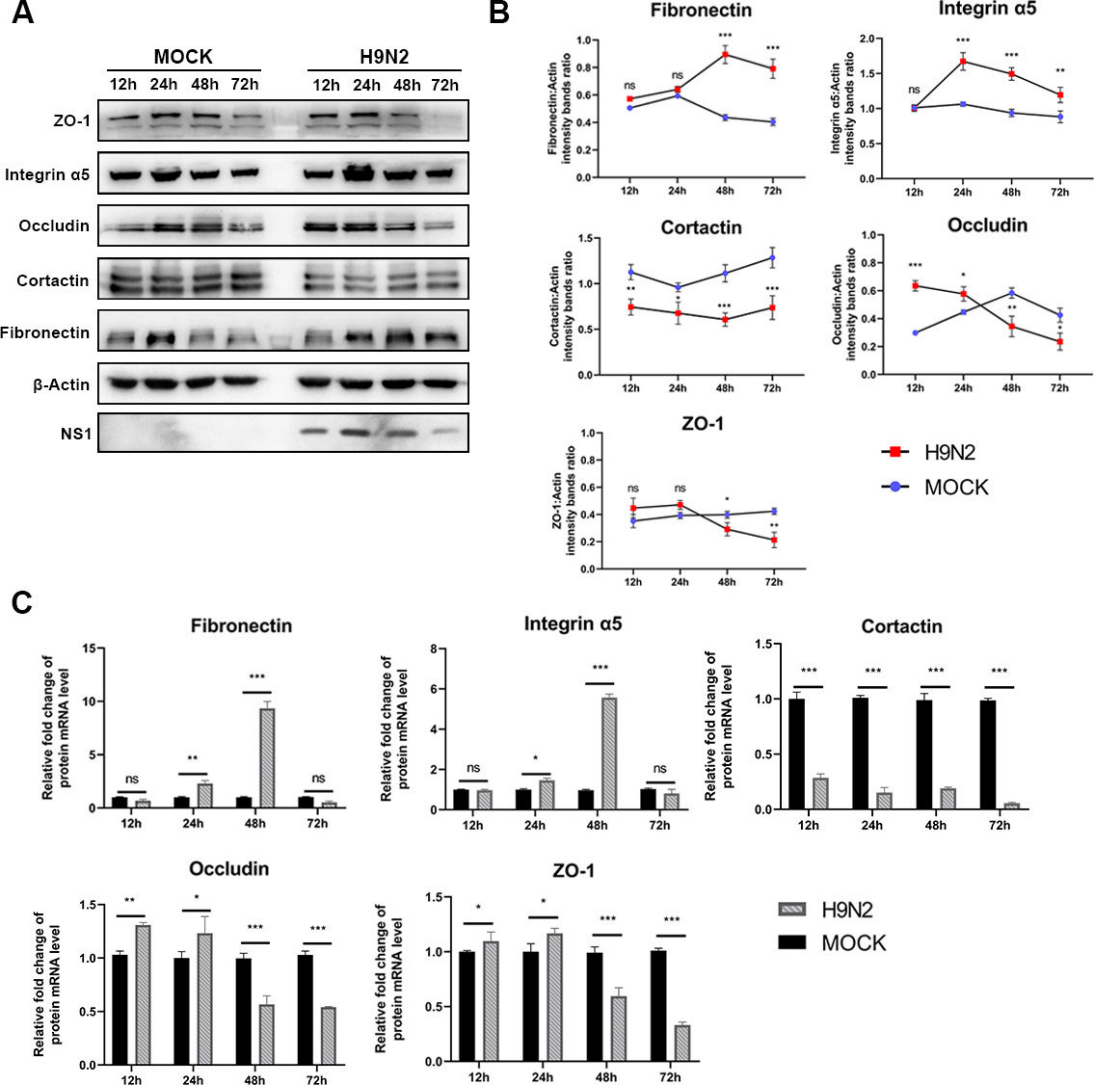
H9N2 infection affects the expression of cellular adhesion molecules. (**A**) Expression of cellular adhesion molecules detected by Western blot. COECs were infected with H9N2 (MOI = 1) for 12, 24, 48 and 72 h, and cellular fibronectin, ZO-1, integrin α5, occludin, and cortactin were determined by immunoblotting. NS1 protein was detected as an indication for virus proliferation, and β-actin was detected for internal control. (**B**) Gray values of the blot were measured to semi-quantify the amount of shown proteins. (**C**) Relative mRNA levels of the genes at different time points post-inoculation examined by quantitative real-time PCR assay. Data are shown as the mean ± standard deviation obtained from three replications. *P < 0.05, **P < 0.01, ***P < 0.001.

Next, potential changes in adhesion molecules on the cell surface were checked to study whether expression of these adhesion molecules was impaired after H9N2 virus infection. Infection of the H9N2 AIV was indicated by virus NS1 protein, and the amount of five cell surface proteins relating to bacteria adhesion, including fibronectin, integrin α5, occludin, cortactin, and ZO-1, was measured by Western blot. Among the checked proteins, fibronectin and integrin α5 showed increased expression after H9N2 infection (Fig. 2A and B). The quantification of the mRNAs of these molecules also supported the WB results, showing upregulated fibronectin and integrin α5 mRNA levels. These results suggest fibronectin and/or integrin α5 may contribute to enhanced APEC adhesion.

### H9N2-enhanced APEC adherence is dependent on the fibronectin protein

**Fig 3:**
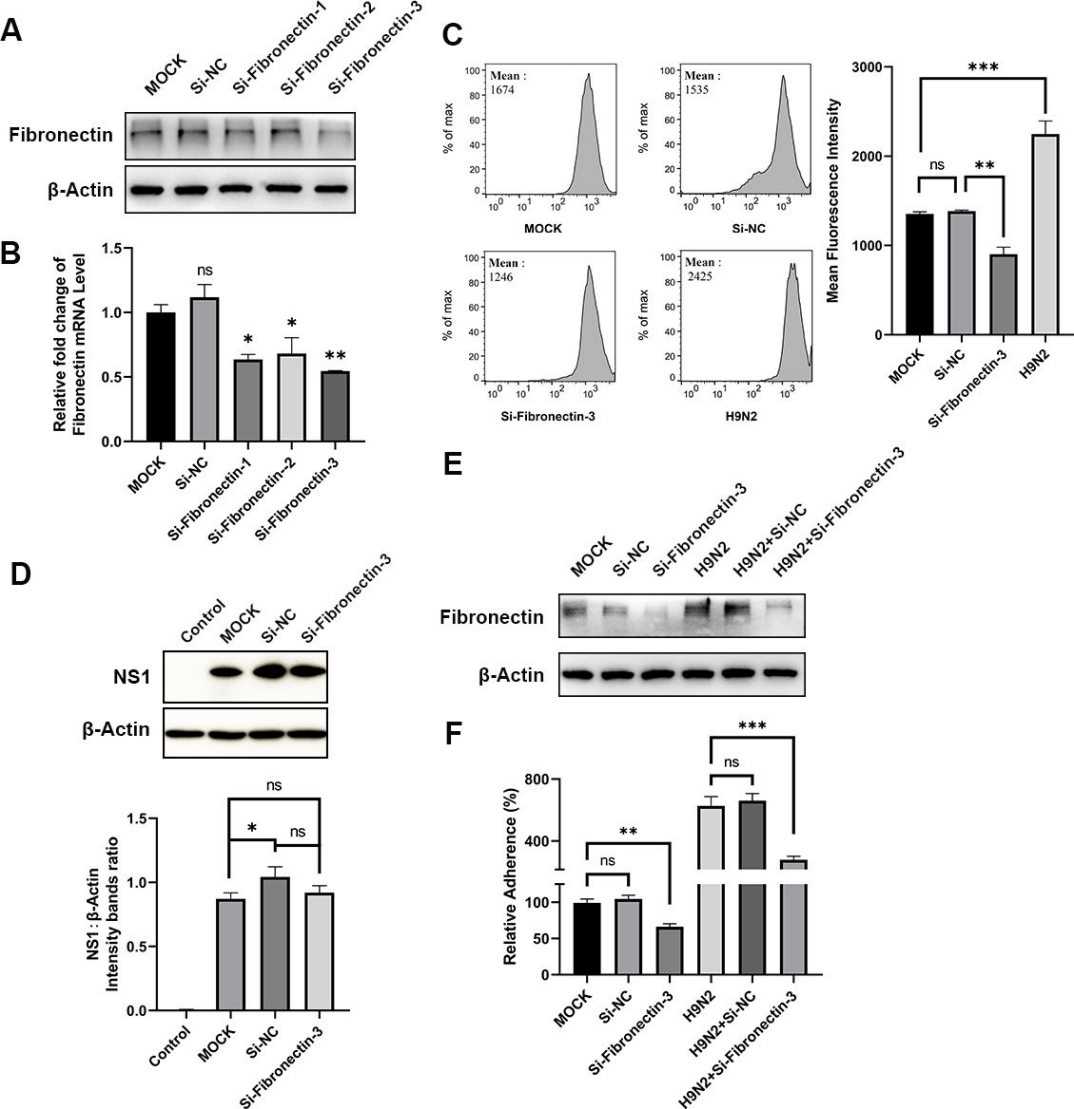
H9N2 AIV-enhanced APEC adhesion is associated with fibronectin upregulation. (**A and B**) Fibronectin protein (**A**) and mRNA (**B**) levels measured by Western blotting and quantitative reverse transcription PCR assay, respectively in COECs treated with three siRNAs. (**C**) Representative flow cytometry histograms of fibronectin fluorescence in COECs after siRNA treatment or H9N2 AIV infection (left) and the average fluorescence intensity of fibronectin from the fluorescence-activated cell sorting assay (right) are shown. Data represent mean ± standard deviation from three independent experiments. (**D**) H9N2 AIV proliferation after fibronectin knockdown. Virus proliferation were indicated by blotting of NS1 protein. (**E**) Fibronectin in COECs after siRNA treatment and H9N2 infection detected by Western blot. (**F**) Relative APEC adhesion measured by plate-counting methods. *P* values were determined by unpaired two-tailed Student’s *t*-test.

Since we have shown that fibronectin expression and APEC adhesion increased after H9N2 infection, we sought to further confirm whether the fibronectin protein is a factor associated with APEC adhesion. Cells were transfected with target-specific short-interfering RNAs (siRNAs) designed to silence the expression of fibronectin, and the gene expression on both protein and mRNA levels was checked by biochemical approaches to test the efficacy of each siRNA. As shown in Fig. 3A and B, all three designed siRNAs could effectively knock down the expression of fibronectin on both protein and mRNA levels. Moreover, one siRNA (short-interfering fibronectin-3), which showed the best efficacy and could reduce cellular fibronectin significantly, was chosen for further applications. Next, COECs were infected with H9N2 or transfected with siRNA, and fibronectin levels were analyzed by flow cytometry, which showed H9N2 infection enhances fibronectin levels and also confirmed that the siRNA could effectively knock down fibronectin (Fig. 3C). To check whether H9N2 AIV-enhanced APEC infection is dependent on fibronectin levels, we used siRNA to suppress the expression of fibronectin protein and infected the cells with H9N2 virus. Quantification of viral protein demonstrated that the H9N2 AIV infection rate was not impaired by fibronectin knockdown (Fig. 3D). Subsequent APEC adhesion experiments showed that the number of adherent APECs decreased upon knocking down the expression of fibronectin using siRNA, and knockdown of fibronectin could reverse elevated APEC adhesion resulting from the virus infection (Fig. 3E and F). Taken together, our data demonstrated that H9N2 virus infection enhanced the level of fibronectin, which is directly related to APEC adhesion in COECs.

### H9N2 infection leads to increased expression of fibronectin in fallopian oviduct tissue

**Fig 4:**
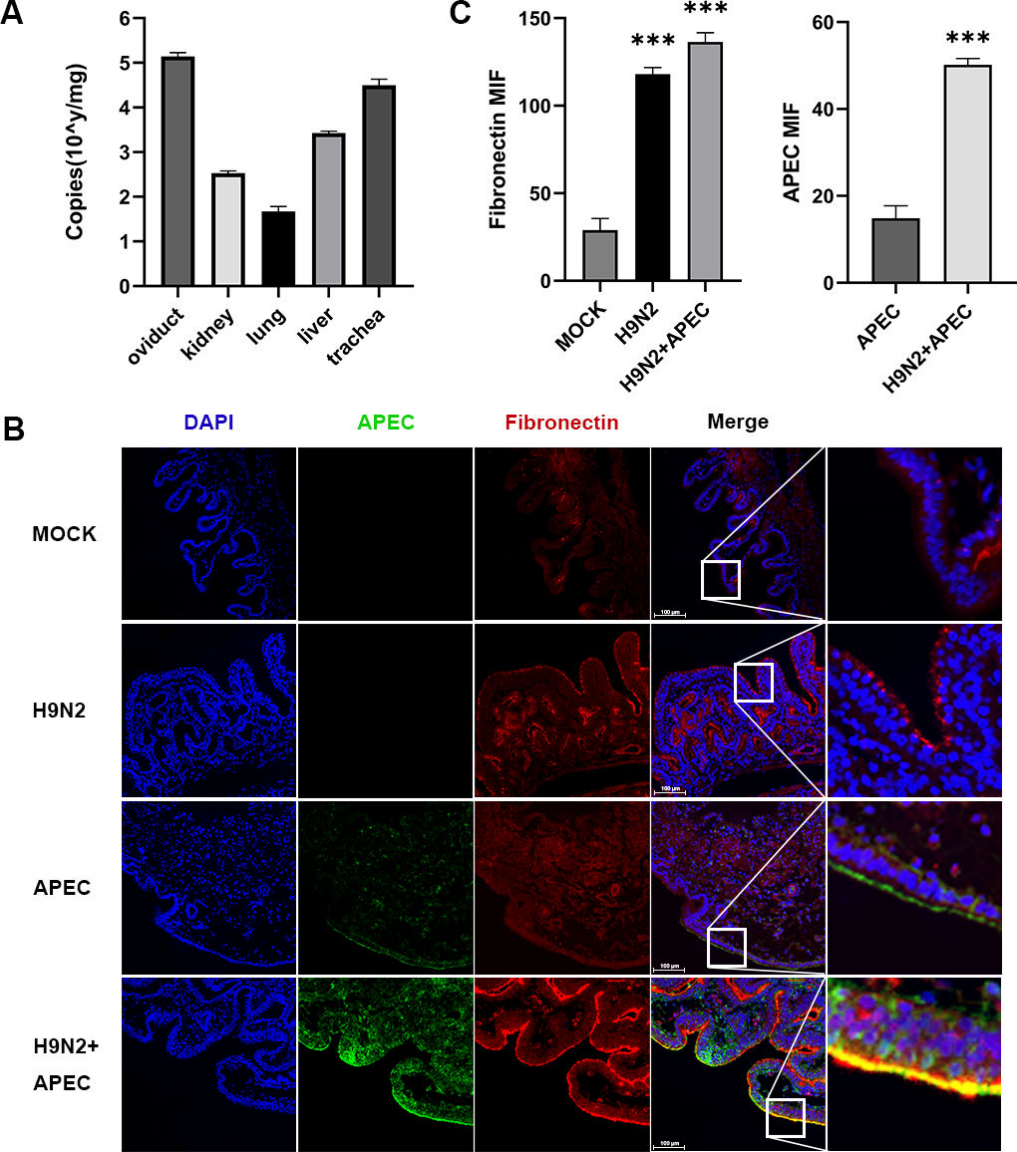
H9N2 AIV increased fibronectin expression and APEC infection in fallopian oviduct tissues. (**A**) Absolute quantitative PCR quantification of H9N2 NS1 transcripts in different tissues after H9N2 virus infection. (**B**) Representative image of fibronectin and infected APEC detected by immunofluorescence in fallopian oviduct tissues. Longitudinal sections are shown, and scale bars represent 100 µm. (**C**) The immunofluorescence intensity of fibronectin protein and APEC at a CFU of 1 × 10^8^ in different groups is expressed by mean fluorescence intensity. Quantified gray values of the fluorescence are presented as means ± standard deviation. The number of samples is 6. Significance of the differences among multiple groups was measured using one-way analysis of variance with Bonferroni’s multiple comparison test. ***P < 0.001. DAPI, 4′,6-diamidino-2-phenylindole.

Next, fibronectin expression and APEC infection after were studied in animals. The study used 160-day-old laying hens as experimental animals, which were divided into four groups, including MOCK, H9N2 infection, APEC infection, and H9N2 plus APEC mixed infection, each consisting of six hens. After infection, we terminated the animal experiment on day 3 because at this time point, the chickens in the experimental group exhibited evident clinical symptoms, typically laying abnormal eggs. Absolute quantification of the virus NS1 transcripts were first performed to identify virus proliferation in different tissues, which revealed varying levels of NS1 protein transcripts in the examined tissues, with the highest amount in oviducts (Fig. 4A). Then, fibronectin levels and APECs in oviducts were further checked by immunofluorescence with antibodies specific for the antigens. H9N2 virus infection leads to elevated amounts of fibronectin on oviduct epithelia, and accordingly, APEC detected on the epithelia cells strongly increased after H9N2 AIV infection (Fig. 4B). Co-localization of APEC and fibronectin fluorescence signals could be observed, indicating potential roles of cellular fibronectin on APEC attachment (Fig. 4B, last row). Quantification of the fluorescence intensities also confirmed H9N2 virus infection increased the fibronectin amount, as well as APEC on the oviduct epithelial cells significantly (Fig. 4C). Our data demonstrated that H9N2 AIV leads to increased expression of fibronectin in fallopian oviduct tissues and therefore enhanced APEC infection.

### H9N2 activates the TGF-β pathway to increase the expression of fibronectin proteins and to increase APEC adhesion

**Fig 5:**
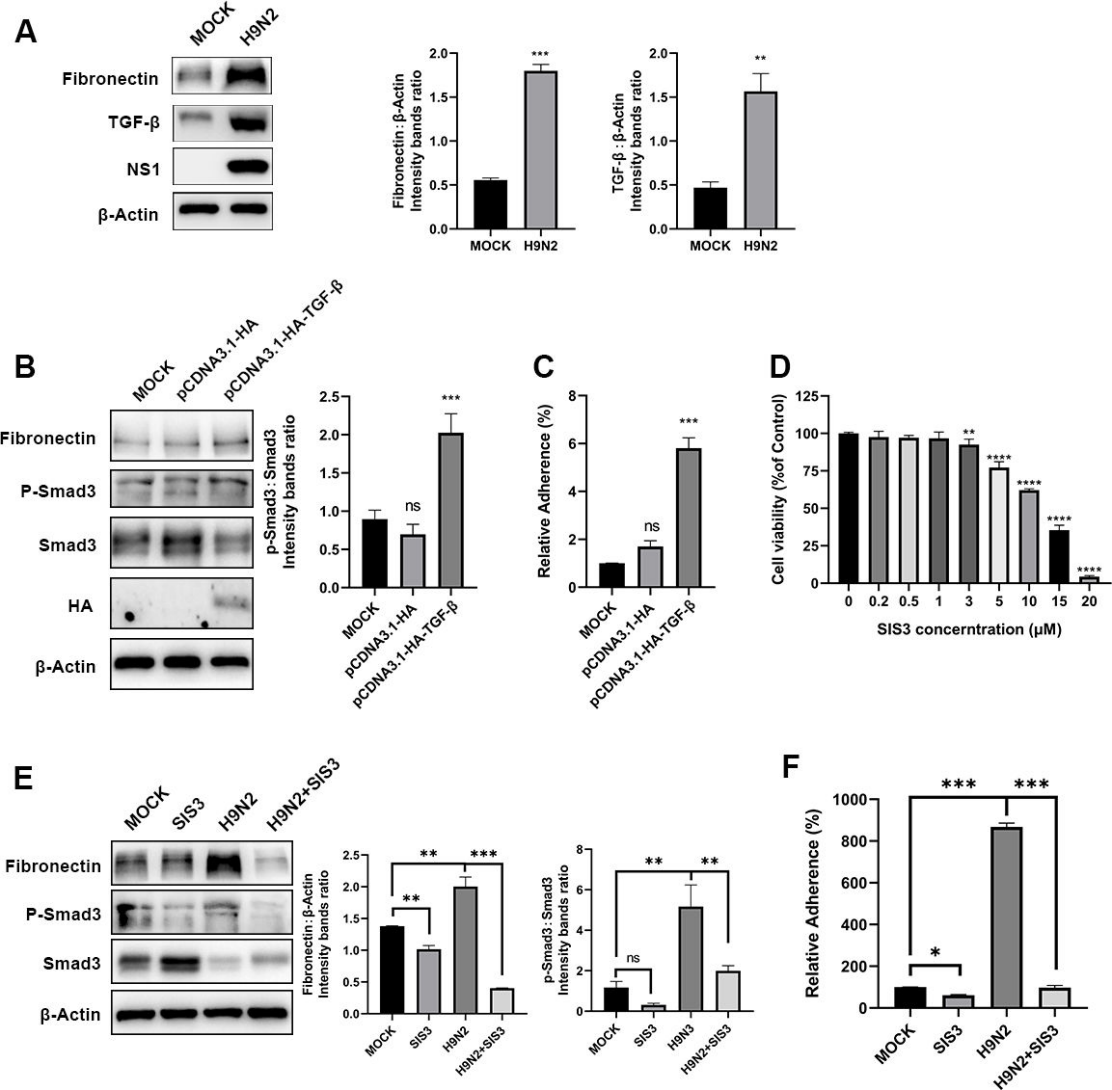
H9N2 AIV infection increases fibronectin expression and APEC adhesion by activating the TGF-β signaling pathway. (**A**) The fibronectin and TGF-β expression in COECs infected with H9N2 (MOI = 1) detected by Western blotting and densitometry analysis. Virus NS1 protein was blotted as an indication of virus infection. Fibronectin and TGF-β levels were semi-quantified by measuring the gray values of the bands and normalized with actin. (**B**) Western blot analysis of fibronectin and p-Smad3 levels in COECs overexpressing TGF-β. The ratio of the phosphorylated and unphosphorylated forms of Smad3 was measured. (**C**) Overexpression of TGF-β promotes APEC (CFU = 1 × 10^8^) adhesion. (**D**) The cell viability after treatment with various concentration of SIS3 checked by CCK-8 assay. (**E**) Changes in fibronectin and TGF-β signaling activation under SIS3 treatment and H9N2 AIV infection. The ratio of p-Smad3 to Smad3 was quantified to measure TGF-β signaling activation. (**F**) Bacteria adhesion to COECs analyzed after inhibition of the TGF-β signaling pathway. Data are representative of three independent experiments (*n* = 6) and presented as mean ± standard deviation for panels. *P < 0.05, **P < 0.01, ***P < 0.001. ns, not significant.

Transforming growth factor beta (TGF-β) promotes fibronectin expression through the TGF-β/Smad signaling pathway (17). Thus, we speculated that the TGF-β/Smad signaling pathway was activated after H9N2 infection and, therefore, increased APEC adhesion through promoted expression of the fibronectin protein. To determine whether TGF-β signaling pathway is activated after H9N2 infection of cells, the cellular TGF-β level was analyzed by immunoblotting. After H9N2 infection, both the TGF-β and fibronectin expression increased significantly in comparison to mock-infected control groups (Fig. 5A), indicating activation of the TGF-β signaling in H9N2 infection. To identify whether activation of the TGF-β/Smad pathway elevated APEC adhesion, we counted the adherent APEC by plate counting in the condition of TGF-β overexpression. COECs were transfected with pCDNA3.1-HA-TGF-β; a significantly higher ratio of phosphorylated SMAD3 (p-SMAD3) to the unphosphorylated form was observed in the TGF-β-overexpressing cells, which indicates activation of TGF-β signaling. Both the fibronectin protein and APEC adhesion increased significantly when TGF-β was overexpressed, verifying the role of TGF-β signaling pathway in fibronectin upregulation and APEC adhesion (Fig. 5B and C).

To further explore the role of TGF-β/Smad signaling pathway in fibronectin activation and APEC adhesion, we used specific inhibitor of Smad3 (SIS3), a TGF-β signaling inhibitor that selectively inhibits the Smad3 molecule, to block the activation of TGF-β signaling. Possible drug-induced cytotoxic effects were assessed by CCK-8 cell viability assay. As shown in Fig. 5D, cells tolerated SIS3 concentrations as high as 5 µM. Then COECs inoculated with H9N2 AIV were treated with 5 µM of SIS3, and fibronectin expression was assessed by Western blot analysis. As shown in Fig. 5E and F, treatment with SIS3 reduced the amount of p-SMAD3 and inverted the upregulation of fibronectin resulting from H9N2 AIV infection. Consequently, adhesion of APEC was inhibited by SIS3 both in H9N2 AIV infected and uninfected conditions. Together, these results proved that the expression of fibronectin and APEC adhesion is upregulated after H9N2 infection and that this upregulation of fibronectin is mediated by activation of the TGF-β signaling pathway.

### The H9N2 NS1 protein interacts with TGF-β to activate the TGF-β pathway

**Fig 6:**
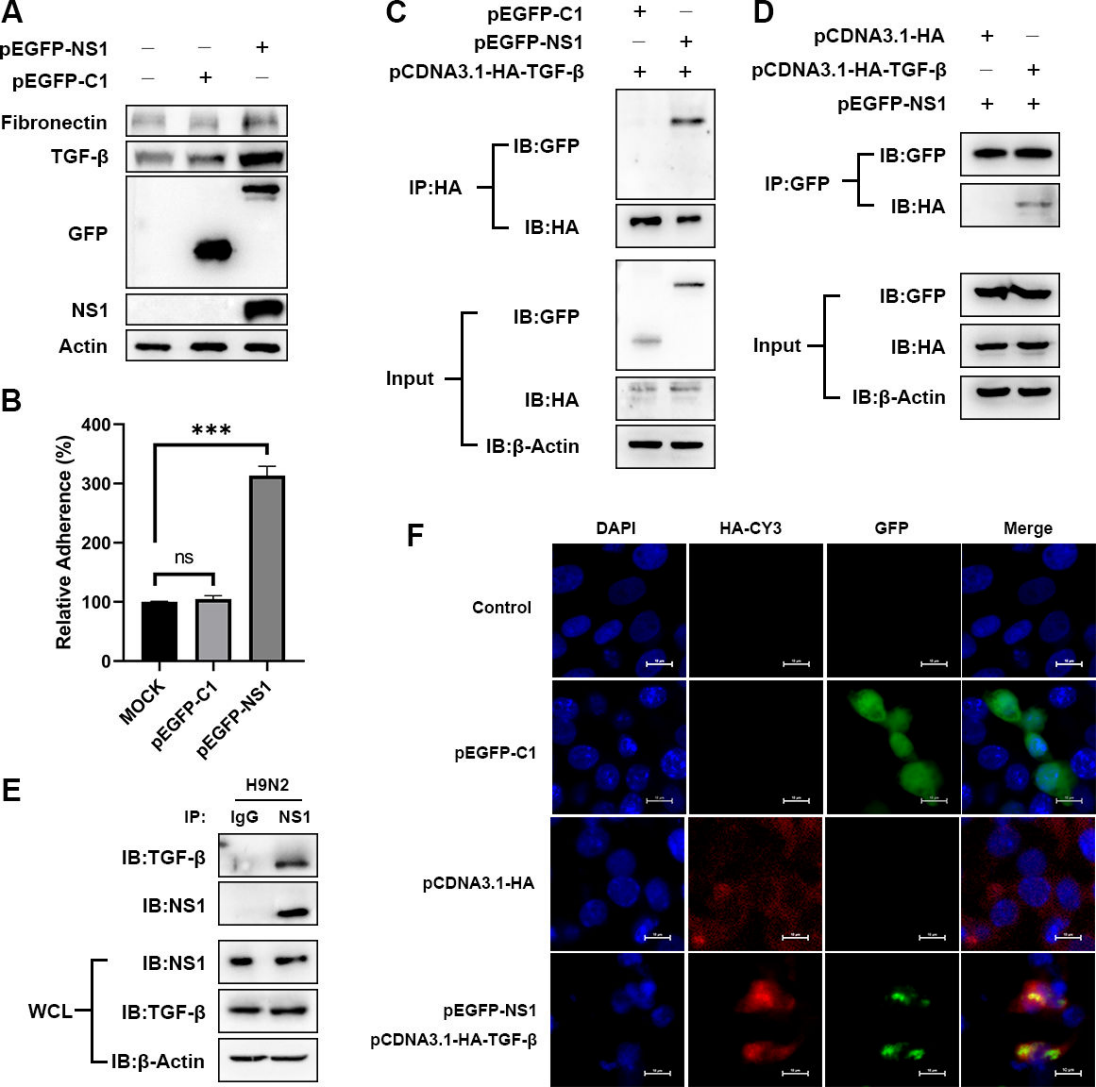
Viral NS1 interacts with and enhances the cellular levels of TGF-β. (**A**) Co-immunoprecipitation of viral NS1 and endogenous TGF-β protein. Lysates from H9N2-infected (MOI = 1) COECs were prepared and immunoprecipitated with the anti-NS1 antibody or control IgG. (**B and C**) Co-immunoprecipitation of recombinant NS1 and TGF-β. 293T cells were co-transfected with pEGFP-NS1 and pCDNA3.1-HA-TGF-β. Cell lysates were subjected to IP with antibodies against HA (**B**) or EGFP (**C**). (**D**) Cellular localization of NS1 and TGF-β in COECs. COECs were transfected with pEGFP-NS1 and/or pCDNA3.1-HA-TGF-β. NS1 and TGF-β were detected by EGFP (green) and anti-HA antibody (red), respectively. Scale bars equal to 10 µm. (**E**) Fibronectin and TGF-β expression levels in cells overexpressing EGFP-NS1 analyzed with Western blot assay. β-Actin was used as a loading control. (**F**) Adhesion APEC to cells overexpressing NS1 counted by plate-counting method. Data are representative of three independent experiments (*n* = 6). Data are presented as mean ± standard deviation for the panels (**F**). ***P  <  0.001. IB, immunoblot; IP, immunoprecipitation; ns, not significant; WCL, whole-cell lysate.

We have previously found that NS1 protein of H9N2 AIV plays important roles in induction of cellular gene expression to facilitate virus proliferation (5, 18). We wondered whether the NS1 protein also participates in the activation of the TGF-β signaling pathway and tested the potential interaction between the two proteins. We infected COECs with H9N2 AIV and performed co-immunoprecipitation (CoIP) experiments using antibodies recognizing viral NS1 protein. Our data showed that NS1 co-immunoprecipitates with the TGF-β protein, indicating an interaction between the two proteins in H9N2 virus infection. In order to further verify the interaction between NS1 protein and TGF-β, plasmids encoding NS1 (pEGFP-NS1) and TGF-β (pCDNA3.1-HA-TGF-β) were transfected into 293T cells, and CoIP experiments were performed with antibodies for immunoprecipitating recombinant NS1 or TGF-β. The results also showed that the NS1 protein co-precipitated with TGF-β on both directions, confirming an interaction between the two proteins (Fig. 6B and C). Furthermore, enhanced green fluorescent protein (EGFP)-tagged NS1 protein also showed co-localization with TGF-β in cells, indicating NS1 played roles in the regulation of TGF-β signaling pathways (Fig. 6D). To further explore the function of NS1 protein in TGF-β signaling, EGFP tagged NS1 protein was overexpressed in cells and subsequently evaluated alterations in the TGF-β signaling pathway as well as the expression of fibronectin. Western blot analysis showed that expression of NS1 enhanced the cellular levels of TGF-β as well as fibronectin. (Fig. 6E). Furthermore, cells overexpressing pEGFP-NS1 were subjected to APEC adhesion assay. Consistent with elevated levels of TGF-β and fibronectin, NS1 protein overexpression group exhibited a significant three- to fourfold increase in APEC adhesion compared to the control group transfected with EGFP plasmid (Fig. 6F). Altogether, our data showed that the NS1 protein of H9N2 AIV interacts with TGF-β and enhances the expression of TGF-β, and therefore promotes downstream fibronectin levels and APEC adhesion to COECs.

## DISCUSSION

Salpingitis is the leading cause of morbidity in egg-producing chickens, resulting in fallopian tube obstruction and decreased egg production (19). Viral and bacterial infections are frequent reasons for chicken salpingitis. H9N2 is a low-pathogenic avian influenza virus that usually induces secondary APEC infection, and co-infection of these two pathogens has a worse effect on chickens than those infected alone (20). Here we discovered H9N2 type AIV infection of COECs promotes APEC infection and further explored the molecular mechanisms behind this phenomenon. Our research applied both *in vitro* COEC model and *in vivo* study to monitor the impact of H9N2 AIV on APEC infection, showing increased APEC adherence and infection after H9N2 AIV proliferation. We found changes in some adhesive molecules on the cell surface of COECs after AIV infection, and these molecules are directly related to APEC adhesions. We further investigated how fibronectin expression is regulated by AIV infection and found that the TGF-β signaling pathway is activated by the NS1 protein of the virus, thus regulating the expression of fibronectin. These results offer new information on how AIV infection promotes APEC secondary infection and provide potential targets for mitigating severe APEC infections induced by H9N2 avian influenza.

The issue of H9N2 infection is highly critical. Particularly noteworthy is the virus’s capability to induce bacterial adherence post-infection, thereby triggering severe symptoms. Influenza virus infection typically leads to grievous pneumonia, and during the development of pneumonia, the elevation of cell adhesion molecules will increase the adhesion of *Streptococcus pneumoniae*, inducing inflammation in the lung (21). A recent study found that H9N2 influenza virus increases *Streptococcus pneumoniae* localization in the lower airway dying epithelial cells and causes severe pneumonia (22). Our study aimed to investigate whether H9N2 infection in the oviduct of hens leads to an increased adhesion of APEC, potentially resulting in severe salpingitis inflammation compared to H9N2 infection alone. Our finding indicates that AIV infection increases APEC adhesion by six- to eightfold following H9N2 infection compared to normal cells, implying a potential association between increased APEC adhesion and more severe salpingitis. The expression of inflammatory factors has not been characterized so far, which should be considered for further investigation in future studies.

Similar to the role of viral receptors on permissive cells, the cell surface molecules might also be critical for bacterial adhesion and infection. The study found that influenza infection increases cell adhesion molecules that promote bacterial adhesion, such as polymeric immunoglobulin receptors has been increased after H1N1 infection in cardiomyocytes (23), and intracellular adhesion molecule 1 expression increased by PR8 infection in A549 cell (24). Our data show that both fibronectin protein and integrin αV protein were found to increase to varying degrees after infection with H9N2 in COECs at different times; these results can also be confirmed by sequencing results after H9N2 infection of the DF1 cell (25). In our research, we studied only the function of fibronectin on APEC adhesion and infection, but whether the other surface molecules, such as integrin αV, or those proteins we have not checked in the study also play roles in APEC infection remains elusive. To answer this question, systematic analysis of the change in cell surface molecules using proteomic and/or transcriptomic assays after AIV infection is required, and with these efforts, the cell surface molecules involving APEC infection and being regulated by H9N2 virus could be identified. This would greatly increase the understanding of mechanisms of APEC co-infection or secondary infection in H9N2 avian influenza.

Notably, while fibronectin and integrin αV protein expressions were upregulated, we also observed that expression of some adhesion molecules such as ZO1 and occludin decreased after H9N2 virus infection. Our finding is consistent with another investigation which studied H1N1 virus infection of alveolar epithelial cells (26). Considering the important roles of ZO1 and occludin in tight junction formation between epithelial cells, decreased levels of these proteins might also contribute to epithelial cell damage and loss of epithelial barrier in chicken oviducts, and therefore, the extracellular matrix (ECM) will be further exposed, resulting to enhanced APEC adhesion in the oviduct.

Prior studies have shown that the cell adhesion molecules of intestinal epithelial cells could interact with colonization factors of *E. coli* to mediate the adhesion of *E. coli* to epithelial cells, and the most important is the ECM constituents (27).

After H9N2 AIV infection in hens, clinical observations often reveal the occurrence of salpingitis, in which the integrity of the oviduct epithelial cells is compromised. This damage leads to an increase in bacterial adhesion, resulting in severe complications. To investigate whether the elevation of APEC adhesion and the alteration of adhesion molecule fibronectin discovered in the *in vitro* model also occur *in vivo*, we conducted animal experiments with H9N2 AIV and APEC infection. Our animal experiments have shown that fibronectin expression is upregulated after H9N2 AIV infection in the oviduct, with fibronectin strongly detected on oviduct epithelial cells. Accordingly, APEC adhesion on oviduct epithelia increases, consistent with the cell model. We have checked the virus load by detecting viral genome copies in different chicken tissues including oviduct, kidney, lung, liver, and trachea after H9N2 AIV infection, which showed that the oviduct has the highest virus load among these tissues (Fig. 4A), further confirming the connection among H9N2 virus infection, fibronectin expression, and APEC adhesion/infection.

The TGF-β pathway is involved in diverse biological and pathological processes and also participates in AIV infection. Previous studies have shown that IAV induces cell damage by phosphorylating JNK1, which in turn produces TGF-β. The TGF-β signaling pathway is the main signaling pathway for activating fibronectin expression (17, 28). For example, influenza virus neuraminidase (NA) can promote increased adhesion of A549 cells through TGF-β-mediated adhesion of adhesion molecules to group A *Streptococcus* and *Lactococcus lactis* (29). Our results demonstrated that the TGF-β signaling pathway is activated by H9N2 virus infection, and fibronectin expression is upregulated upon TGF-β activation. In this study, we revealed the role of the TGF-β pathway in APEC infection, which provides potential targets for APEC secondary infection prevention. Inhibition of the activation of TGF-β signaling pathway or blocking the binding of fibronectin to APEC may contribute to control of APEC infections.

The influenza virus harbors a diverse array of viral proteins, each fulfilling roles in the intricate process of viral infection. Previous studies in our laboratory suggested that the NS1 protein plays an important role in H9N2 infection of COECs, including causing apoptosis (18). The NS1 protein of the influenza virus acts as an independent regulator directly involved in controlling the severity of secondary bacterial infections by regulating the IFN-β response, as described in the research result (30). The above studies suggest that the NS1 protein is important for viral infection of cells, and our results confirm this interaction affecting fibronectin expression. In the present study, H9N2 NS1 is shown to interact with TGF-β and play an important role in activating TGF-β signaling and increasing APEC adhesion. While our experiments specifically focus on the NS1 protein of the H9N2 virus and its role in TGF-β activation, there is research on the activation of the TGF-β pathway by the NA of PR8 virus (29). It would be interesting to explore whether the activation of TGF-β pathway by NS1 and/or NA protein is subtype specific or a commonly used mechanism in all types of AIV infections.

Here, we showed the low-pathogenic H9N2 subtype AIV infection activates TGF-β signaling pathway, thus upregulating the amount of cellular fibronectin and facilitating APEC infection. It is worth exploring whether other subtypes of AIV possess a similar mechanism for secondary bacterial infection in other species. Studies on the PR8 influenza virus (an H1N1 subtype AIV) showed that the TGF-β signaling pathway is also activated by virus infection (29); proteins on the cell surface such as integrin αV and fibronectin are upregulated, consistent with our current study. Besides, a recent study focusing on small RNA sequencing reported that the expression of miRNAs targeting to the TGF-β signaling pathway in chicken trachea is significantly regulated after H5N1 virus infection, also supporting potential roles of the TGF-β signaling pathway in H5N1 subtype AIV infection (31). Based on this research on other subtypes of AIV as well as our own unpublished data, we speculate that the mechanism discovered in this study is not exclusive for the H9N2 subtype and may also exist in both low- and high-pathogenic subtypes of AIV.

In conclusion, we provide evidence that H9N2 infection of COECs activates TGF-β signaling through the interaction of NS1 and TGF-β, increases fibronectin expression and APEC adhesion, and leads to more severe salpingitis. In the aforementioned experiments, the study focused on elucidating how H9N2 promotes APEC infection, but the specific functions of individual viral proteins of H9N2 were not explored. Therefore, intentional investigation of the distinct roles of each viral protein was undertaken. The obtained research findings carry insights for controlling bacterial secondary infections arising from other types of low-pathogenic avian influenza.

## MATERIALS AND METHODS

### Cell culture and virus infection

Primary COECs were obtained as previously described, and cell purity was validated prior to testing by detecting cytokeratin expression (18, 32, 33). The cells were cultured in Dulbecco’s minimal essential medium/nutrient mixture F-12 Ham’s medium (DMEM/F12) supplemented with 10% fetal bovine serum (FBS), 2% heat-inactivated chicken serum, penicillin (100 IU/mL), streptomycin (10 µg/mL), epidermal growth factor(10 ng/mL), insulin (0.12 U/mL), and estradiol (50 nM) at 37°C under 5% CO_2_. H9N2 subtype AIV strain (A/chicken/Shaanxi/01/2011) was obtained from infected chicken in Shaanxi, China, and reproduced in 10-day-old embryonated chicken eggs at 37°C for 72 h, as previously reported (34).

When the COECs were 80%–90% confluent, they were infected with H9N2 AIV (A/chicken/Shaanxi/01/2011) at an MOI of 1 or mock infected with sterile phosphate-buffered saline (PBS), as previously reported (18, 33).

Aspiration was used to remove unattached viruses after a 1.5-h adsorption period. The cells were then rinsed three times with sterile PBS before being cultivated for varied time periods in fresh DMEM/F12 supplemented with 2% FBS, and 2-µg/mL Tosyl-L-phenylalaninchloromethylketon (TPCK) treated trypsin was added during the culture process.

### Virus

The H9N2 used was a strain clinically isolated in 2012, and the sequencing information has been published (35). H9N2 viruses were propagated in chicken embryos using standard procedures. Specifically, 10-day-old specific pathogen-free chicken embryos were infected with H9N2 IAV and incubated at 33°C and 60% humidity for 48 h, followed by overnight incubation at 4°C. Allantoic fluid was collected, aliquoted, and stored at −80°C.

### 50% tissue culture infectious dose (TCID_50_) assay

The total virus yields were quantified using the TCID_50_ assay based on the Spearman–Kärber method and our previously published study (32, 36). In brief, COECs in 96-well plates were exposed to H9N2 AIV dilutions via a 10-fold serial dilution. After a 1-h incubation at 37°C, the culture supernatants were replaced with DMEM containing 2% FBS. Cells were cultured for 5 days, and the number of wells with cytopathic effects were recorded and virus titers were determined by the Spearman–Kärber method.

### APEC adhesion and counting

*Escherichia coli* was preserved in this laboratory. This pathogenic strain was obtained from hens with salpingitis illness and kept in glycerol at −80°C. The strain was resuspended on lysogeny broth (LB) and cultured at 37°C overnight before being stored at 4°C until use. Determination of CFU was done by plate counting on LB agar plate. Briefly, 1 mL of APEC bacterial solution was diluted 10-fold serially with PBS and plated onto tryptic soy agar, and finally, the colonies were counted after 24 h of incubation at 37°C. The bacteria was added to the cells at 37°C for 2 h. Unbounded bacteria were removed with PBS, and cells as well as adherent bacteria were collected, then APEC was counted with plate counting.

### Cell viability assay

The Cell Counting Kit-8 (CCK-8) test was used to determine the cytotoxicity of SIS3 (Beyotime, Shanghai, China). COECs were seeded in 96-well plates and cultured at 37°C in 5% CO_2_; SIS3 was introduced at a certain concentration; and the cells were cultivated for another 48 h. Then, following the manufacturer’s instructions, 10-µL CCK-8 reagent was applied to each well of a 96-well plate containing 100-µL culture media and was incubated for 2 h at 37°C. Absorbance measurements at 450 nm were used to determine cell viability. The viability was represented as the percentage of the optical density of wells in comparison to wells containing untreated control cells that were regarded as 100% viable.

### Quantitative reverse transcription PCR

The experimental procedures were conducted with reference to the methods in previously published papers (37). Total RNA from cells was extracted using the TRIzol reagent (TransGen Biotech, Beijing, China) and reverse transcribed into cDNA using Easy Script One-Step RT-PCR SuperMix (TransGen Biotech) according to the manufacturer’s instructions. Quantitative PCR (qPCR) reaction was performed on the Step One Plus real-time PCR system (Applied Biosystems, CA, USA) using FastStart Universal SYBR green master (TransGen Biotech). Primers were designed using the National Center for Biotechnology Information primer designing tool. All primers were synthesized by Sangon Biotech Corporation (Beijing, China), and the sequences of the primers are listed in the Table 1 below. The 2^−ΔΔCT^ method was used to calculate relative expression, which was then normalized by levels of β-actin. For quantification, samples from at least three different passes were used.

**TABLE 1:** Primer sequences for the relevant genes’ amplification during reverse transcription PCRs

Gene	Primer	Sequences (5′→3′)
β-Actin	Forward	AGACATCAGGGTGTGATGGTTGGT
	Reverse	TGGTGACAATACCGTGTTCAATGG
Fibronectin	Forward	CACTGGTTGGCCGAAAAAGG
	Reverse	TGCCTGGAAGTTGGATACCG
Cortactin	Forward	AGGGTGTGGAATTAGCAGCAG
	Reverse	TTCACTCACTGGTAGCACCC
Integrin αV	Forward	ATTCTGGCTTGTGCCCCATT
	Reverse	AGCAGGACTCTGTCTCCCTT
ZO-1	Forward	GCCTGAATCAAACCCAGCAA
	Reverse	TATGCGCGGTAAGGATGAT
Occludin	Forward	ACGGCAGCACCTACCTCAA
	Reverse	GGGCGAAGAAGCAGATGAG

### Western bolt analysis

As previously mentioned, protein homogenates from the oviduct epithelial cells were isolated. In essence, the cells were lysed with RIPA buffer (Solarbio, Beijing, China) mixed with protease inhibitor phenylmethylsulfonyl fluoride (PMSF) (Solarbio, Beijing, China) for 20 min on ice. The bicinchoninic acid (BCA) protein assay kit (Beyotime) was utilized to determine the protein content of each sample. Following that, identical amounts of protein were separated on 8%–15% SDS-polyacrylamide gel and then transferred to polyvinylidene difluoride membranes (Millipore, Billerica, MA, USA). The membrane was blocked with 5% skim milk (Thermo Scientific) in tris-buffered saline with Tween 20 (TBST) for 2 h, and then the membrane was incubated with different primary antibodies overnight at 4°C. Anti-β-actin primary antibody was obtained from TransGen Biotech. AIV NS1, Smad3, phospho-Smad3, and occludin primary antibody were purchased from Santa Cruz Biotechnology (CA, USA). Fibronectin, TGF-β, and E-cadherin primary antibody were purchased from Abcam (Cambridge, UK). Integrin αV, ZO-1, and cortactin primary antibody were purchased from Genetex (CA, USA). HA and GFP tag antibody were purchased from Cell Signaling Technology (Boston, MA, USA). After washing the membrane with TBST, the HRP-conjugated goat anti-rabbit secondary antibody or the HRP-conjugated goat anti-mouse secondary antibodies (both from TransGen Biotech) were applied for 1.5 h at room temperature. Using enhanced chemiluminescence immunoblotting detection reagents, we checked the bound antibodies (Millipore). A CanoScan LiDE 100 scanner (Canon) was used to capture the images. Quantification of the blot was performed with ImageJ (version 1.53c). The intensity of the band was measured by the total gray value of the band with background subtraction and then normalized by the intensity of β-actin of each lane. The Western blot results were repeated at least three times, and the average intensity, as well as the standard deviation of the band intensity, was calculated and shown as histogram.

### CoIP assays

pcDNA3.1-HA-TGF-β and pEGFP-NS1 plasmids were transfected into DF1 cells. At 48 h after transfection, the cells were lysed with cell lysis buffer in the presence of protease inhibitor (Beyotime) for 30 min at 4°C, then centrifuged at 12,000 × *g* for 10 min. Then, the supernatant was collected and mixed with 1 µg of anti-HA or anti-GFP antibody. The sample was incubated overnight at 4°C then coupled with protein A + G agarose beads (Santa Cruz Biotechnology, sc-2003, USA) at 4°C for 6 h and centrifuged, and the agarose beads were collected. The beads were washed three times with pre-cooled PBST. Finally, the immunoprecipitated protein was extracted from the agarose beads by boiling for 10 min in 5 × SDS-PAGE sample buffer, and the bound protein was analyzed by Western blot.

### Scanning electron microscopy

*Escherichia coli* was grown in lysogeny broth for 16 h at 37°C and counted using the usual plate-counting method. Cells were seeded in 24-well plates placed with coverslips, infected for 48 h with 1 MOI H9N2 AIV, and washed three times with PBS. Cells were infected with *E. coli* for 2 h for adherence. Cells were washed three times with PBS to eliminate non-adherent *E. coli*, then fixed with 4% paraformaldehyde (PFA).

Thereafter, samples were either processed for SEM immediately or stored at 4°C until later use. Cell samples were washed three times in water for 5 min each time, dehydrated with series gradient alcohol, 30%, 50%, 70%, 80%, 90%, 95%, and 100%, 10 min per gradient. The slide was gently glued to the conductive adhesive, and the ion sputter (E-1045; Hitachi, Japan) was sprayed. The suitable areas were selected under a scanning electron microscope (Inspect, FEI, USA), and digital images were acquired with appropriate magnifications.

### Flow cytometry

Cells were trypsinized and spun down at 200 × *g* for 5 min. Next, cells were resuspended and adjusted to 1 million/mL. Then an equal volume of 1% PFA was added at 4°C for 15 min followed by washing with PBS. Then 0.15% Triton X-100 solution was added to permeabilize cell membranes followed by washing with PBS.

To avoid non-specific binding, 1 × 10^6^ cells were first pre-incubated with PBS containing 3% bovine serum albumin (BSA) (Sigma–Aldrich GmbH, Steinheim, Germany) for 30 min at room temperature. Thereafter, cells were washed with 0.5% BSA (in PBS) and incubated with saturating amounts of monoclonal antibodies against the fibronectin in separate tubes for 1 h at 37°C. After a washing step, the samples were incubated with AlexaFluor 488-conjugated goat anti-rabbit IgG Abs (33106ES60; Yeasen, Shanghai, China) for 1 h at 37°C. Labeled cells were resuspended in 200-µL PBS and stored at 4°C before flow cytometric analysis. The optimal dilution of each antibody was determined prior to the experiments, and the same batches of antibody were used throughout the study. Fluorescence-activated cell sorting was used to analyze the samples (FACSCalibur, BD, USA). Three independent experiments were performed for statistical analysis. FlowJo software was used to visualize the data (Tree Star, Ashland, OR, USA).

### Vector construction and plasmids, siRNAs transfection

The TGF-β gene (GenBank No. NM_001318456.1) was amplified from the cDNA of oviduct of chicken by reverse transcription PCR and then subcloned into the pcDNA3.1 vector to generate pcDNA3.1-HA-TGF-β. The pEGFP-NS1 was constructed and conserved by our laboratory. Pooled and validated siRNAs targeting fibronectin were purchased from RiboBio (Guangzhou, China). Before transfection, cells were cultured in 12-well plates. When reaching approximately 70% confluence, the cells were transfected with 2-µg specific plasmid or 100-nM siRNA, using empty plasmid pcDNA3.1 or SiNC as negative control. Twenty-four hours after transfection, the cells were infected with H9N2 AIV and further cultured for 48 h then infected with APEC.

### Confocal fluorescence microscopy

pCDNA3.1-HA-TGF-β and pEGFP-NS1 plasmids or mock plasmids were transfected into COEC cells. Forty-eight hours after transfection, the cells were fixed with 4% paraformaldehyde for 15 min, treated with 0.5% Triton X-100 for 5 min, and then blocked with 5% BSA for 2 h. Anti-HA antibodies were incubated in a 4°C incubator overnight, washed with phosphate-buffered saline with Tween (PBST) four times, mixed with AlexaFluor-594-labeled goat anti-rabbit IgG (dilution ratio: 1:200), incubated at 37°C for 1.5 h, stained with Hoechst 33258 (Yeasen) at room temperature for 10 min, and observed under a laser confocal scanning microscope (Carl Zeiss AG, Oberkochen, Germany).

### Immunofluorescence

Chickens were anesthetized with 10% chloral hydrate with the oviduct tissues fixed with 4% PFA overnight and then embedded in paraffin wax. Four-millimeter sections were cut and dewaxed in xylene twice for 15 min, followed by gradual rehydration with 100%, 85%, and 75% gradient alcohol and distilled water for 5 min. A microwave oven was used for antigen repair for 15 min on tissue sections placed in a repair box filled with EDTA antigen repair buffer (pH 8.0). After cooling to room temperature, the sections were washed three times, 5 min each time, with PBS (pH 7.4). The ring was filled with a spontaneous fluorescence quenching agent for 5 min and then washed with flowing water for 10 min. The sections were blocked with bovine serum albumin for 30 min and incubated with primary antibodies in PBS at 4°C overnight. The primary antibodies were as follows: rabbit anti-fibronectin antibody 1:200 (Abcam) and rabbit anti-*E*. *coli* monoclonal antibody 1:200 (Genetex, Taiwan, China). After three washes with PBS for 5 min each, the sections were incubated with fluorophore-labeled antibodies [CY3 goat anti-rabbit IgG (Servicebio, Wuhan, China; GB21303); fluorescein isothiocyanate (FITC) labeled goat anti-rabbit IgG (Servicebio, GB22303)] at room temperature for 50  min in the dark. Finally, the slides were counterstained with 4′,6-diamidino-2-phenylindole at room temperature for 10 min, sealed with an anti-fluorescence quencher, and visualized under a fluorescence microscope (Nikon Eclipse C1, Japan); the images were captured by Nikon DS-U3.

### Chicken experiments

At the age of 160 days, female chickens, verified to be seronegative for presently circulating H9N2 strains through a conventional hemagglutination assay, were selected for the study. In each experimental group, six chickens underwent anesthesia with vaporized isoflurane, after which they were intranasally administered with 10^8^ viral particles of the designated A (H9N2) strains and suspended in a 1-mL volume, and 0.2-mL virus was inoculated for each hen. Subsequently, each group underwent daily monitoring to detect any manifestations of infection. It was on the third day post-inoculation when obvious clinical symptoms, such as the production of abnormal eggs, were observed, and euthanasia procedures were then performed. Following euthanasia, oviduct tissues were meticulously extracted and subsequently subjected to fixation using a 4% paraformaldehyde solution, followed by paraffin embedding. Thereafter, the oviduct tissue sections underwent immunofluorescent staining for further analysis.

For NS1 gene quantification, multiple tissues of hens were sampled (including oviduct, kidney, lung, liver, and trachea) and ready for RNA extraction. Total RNA from the collected samples was extracted using Trizol reagent, and NS1 cDNA was synthesized and quantified as previously described in the quantitative reverse transcription PCR section. The absolute copy numbers of the NS1 gene in the samples were calculated using linear regression of the standard curve. pEGFP-NS1 plasmid starting at 10^9^ copies/μL was serially diluted with DNase/RNase-free water and quantified by qPCR, and the CT values were recorded for generation of standard curve by linear modeling of the given plasmid DNA concentration and CT values.

## Data Availability

The authors confirm that the data supporting the findings of this study are available within the article.

## AFTER EPUB

*[This article was published on 28 February 2024 with an error in the abstract. The abstract was corrected in the current version, posted on 19 March 2024.]*
